# Poster Session I - A159 IMPACT OF GLP-1 RECEPTOR AGONISTS ON BOWEL PREPARATION QUALITY IN PATIENTS UNDERGOING ELECTIVE COLONOSCOPY

**DOI:** 10.1093/jcag/gwaf042.159

**Published:** 2026-02-13

**Authors:** P Gaulin, G Major, M Lamontagne, M Bureau, J Dion

**Affiliations:** Gastroentérologie, Hotel-Dieu de Sherbrooke, Sherbrooke, QC, Canada; Hotel-Dieu de Sherbrooke, Sherbrooke, QC, Canada; Hotel-Dieu de Sherbrooke, Sherbrooke, QC, Canada; Gastroentérologie, Hotel-Dieu de Sherbrooke, Sherbrooke, QC, Canada; Hotel-Dieu de Sherbrooke, Sherbrooke, QC, Canada

## Abstract

**Background:**

Glucagon-like peptide-1 receptor agonists (GLP-1RA) are increasingly prescribed for diabetes and obesity but are known to slow gastrointestinal motility. Data on their impact on bowel preparation quality before colonoscopy is conflicting, and no specific recommendations currently exist regarding bowel preparation in GLP-1RA users.

**Aims:**

This study aimed to assess whether GLP-1RA use affects bowel preparation quality measured by the Boston Bowel Preparation Scale (BBPS) in patients undergoing elective colonoscopy. A secondary objective was to explore potential interaction with diabetes status as diabetes itself is associated with gastrointestinal dismotility.

**Methods:**

We conducted a retrospective cohort study of 1,419 adults who underwent elective colonoscopy at a tertiary academic center in 2023. Patients actively taking a GLP-1RA at the time of colonoscopy (no recommendation to stop the medication was made) were compared with non-users. Exclusion criteria included bowel motility disorders, use of prokinetics, or prior colonic surgery. The primary outcome was bowel preparation quality measured by BBPS, analyzed as both continuous and dichotomized (inadequate preparation defined as total BBPS <7. Logistic regression assessed associations between GLP-1RA use and inadequate preparation, with multivariable analysis adjusting for age, sex, diabetes, body mass index, and laxative use.

**Results:**

Forty-one patients (3.0%) used GLP-1RA. They had higher BMI (34.2 ± 7.3 vs 27.8 ± 6.0, p < 0.001) and were more frequently diabetic (78% vs 16.5%, p < 0.001). BBPS was documented in 99.6% of colonoscopies. Mean BBPS was lower among GLP-1RA users (7.7 ± 1.9 vs 8.5 ± 1.4, p < 0.001), repeat colonoscopy due to inadequate preparation was more common (22% vs 9%, p = 0.014). In univariate analysis, GLP-1RA use (OR 2.7 [1.2–5.6], p = 0.01), diabetes (OR 2.6 [1.8–3.8], p < 0.001), and laxative use (OR 5.2 [3.3–8.0], p < 0.001) were associated with inadequate preparation. However, in the multivariable model including both GLP-1RA use and diabetes, only diabetes remained significant (OR 2.4 [1.6–3.6], p < 0.001), while GLP-1RA use was no longer associated with inadequate preparation (1.6 [0.7–3.5], p = 0.24). The overall adenoma detection rate (ADR) was 35.1%.

**Conclusions:**

GLP-1RA use was statistically associated with poorer quality of bowel preparation and a more frequent need to repeat the colonoscopy, which is highly clinically significant. However, this effect appears largely attributable to coexisting diabetes which emerged as the main predictor of inadequate bowel preparation. Prospective studies are needed to better delineate the respective contributions of diabetes and GLP-1RA use to bowel preparation quality and to determine whether tailored preparation protocols are warranted for diabetic and/or GLP-1RA using patients.

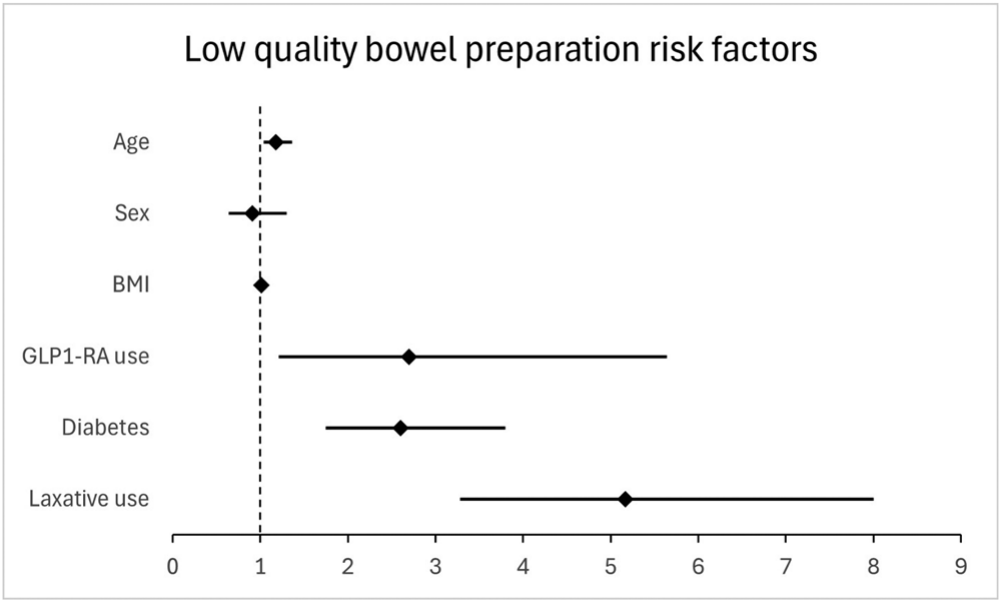

**Funding Agencies:**

None

